# Blockade of CXXC5-dishevelled interaction inhibits adipogenic differentiation, obesity, and insulin resistance in mice

**DOI:** 10.1038/s41598-022-25315-x

**Published:** 2022-11-30

**Authors:** Seol Hwa Seo, Dasung Lee, Soung-Hoon Lee, Kang-Yell Choi

**Affiliations:** 1 Department of Biotechnology, College of Life Science and Biotechnology, Yonsei University, Seoul, 03722 Republic of Korea; 2 CK Regeon Inc, Seoul, 03722 Republic of Korea

**Keywords:** Biotechnology, Metabolomics, Metabolic disorders

## Abstract

Obesity has become a major risk factor for developing metabolic diseases, including insulin resistance, type 2 diabetes, and hypertension. Growing pieces of evidence indicate that the Wnt/β-catenin signaling pathway plays an important role in adipogenesis and obesity. Activation of the Wnt/β-catenin signaling pathway inhibits adipogenesis by suppressing the differentiation of committed preadipocytes into mature adipocytes. CXXC5 is highly induced with suppression of Wnt/β-catenin signaling in early adipogenic differentiation. In addition, silencing CXXC5 in vitro increased β-catenin and decremented the major adipogenic differentiation markers. KY19334, a small molecule that activates the Wnt/β-catenin pathway via inhibition of CXXC5- Dishevelled (Dvl) protein–protein interaction (PPI), suppressed adipogenic differentiation. Administration of KY19334 ameliorated obesity by 26 ± 1.3% and insulin resistance by 23.45 ± 7.09% and reduced adipocyte hypertrophy by 80.87 ± 5.30% in high-fat diet (HFD)-fed mice. In addition, KY19334 accelerated the browning of adipose tissue and promoted hepatic glucose homeostasis in HFD-fed mice. In conclusion, activation of the Wnt/β-catenin signaling by inhibiting the interaction of CXXC5 and Dvl by small molecule-mediated interference is a potential therapeutic approach for treating obesity and insulin resistance.

## Introduction

Obesity has become a global epidemic and is associated with metabolic diseases, including type 2 diabetes, dyslipidemia, and hypertension, and is also known as a high-risk factor severe COVID-19^1,2^. In the past, dietary and lifestyle modifications can be effective to in improving obesity and preventing diabetes. However, these approaches are challenging to maintain in the long term^3^. Current pharmacological approaches for treating obesity focus on reducing appetite, calorie intake, or absorption of dietary fat^4–6^. Unfortunately, anti-obesity therapies have been unsatisfactory due to the adverse effects, low safety, and poor long-term maintenance of weight loss^6–8^. Therefore, understanding the regulatory mechanisms of adipogenesis is necessary to develop better therapies for treating obesity and adipose tissue inflammation.

The canonical Wnt/β-catenin pathway plays important roles in the development, tissue homeostasis, cell proliferation, and cell differentiation^9,10^. Activation of the Wnt/β-catenin pathway inhibits adipogenesis by suppressing the expression of the peroxisome proliferator-activated receptor gamma (PPARγ) and CCAAT/enhancer-binding protein alpha (C/EBPα)^9,10^ which are subjected to the repression by WNT1-inducible signaling pathway protein-1 (WISP1), a direct transcriptional target of the Wnt/β-catenin signaling. Therefore, identifying a factor regulating the Wnt/β-catenin pathway, especially which drives obesity, is important for developing anti-obesity drugs.

CXXC5-type zinc finger protein 5 (CXXC5) is a negative feedback regulator of the Wnt/β-catenin pathway that functions via binding to Dishevelled (Dvl)^11,12^. CXXC5 plays various pathophysiological roles involving adult tissue regeneration at the specific pathophysiological status^13–16^. Growing evidence indicates that CXXC5 is a key driving factor for metabolic diseases^17^ and inhibition of CXXC5 function accelerates liver tissue regeneration with metabolic improvements^18^. Thus we reasoned that CXXC5 might be highly expressed with suppression of Wnt/β-catenin signaling in the early adipogenic differentiation during the development of obesity. Here, we investigated the role of the CXXC5 in inhibiting Wnt/β-catenin signaling and adipocyte differentiation by using KY19334, a small molecule that activates the Wnt/β-catenin signaling via interference in the CXXC5-Dvl interaction^19^. By oral administration, KY19334 improved the pathological features of obesity, including adipocyte hypertrophy and hepatic steatosis induced by a high-fat diet (HFD). The systemic effectiveness of KY19334 on the anti-obesity was acquired by blockade of the function of the aberrantly overexpressed CXXC5 and subsequent inhibition of the Wnt/β-catenin signaling suppression in the early developmental stage of obesity by HFD. Indirect activation of Wnt/β-catenin signaling, actually its restorative activation by the interference of the CXXC5 function, could be an effective and safe approach for anti-obesity.

Taken together, our findings revealed that the small molecular approach for activating Wnt/β-catenin signaling via interference of the CXXC5-Dvl protein–protein interaction (PPI) provides a potential anti-obesity approach against over-nutrition involving HFD.

## Results

### The Cxxc5 plays a role in the adipocyte differentiation of 3T3-L1 preadipocytes by suppressing Wnt/β-catenin signaling via binding Dvl

**Figure 1:**
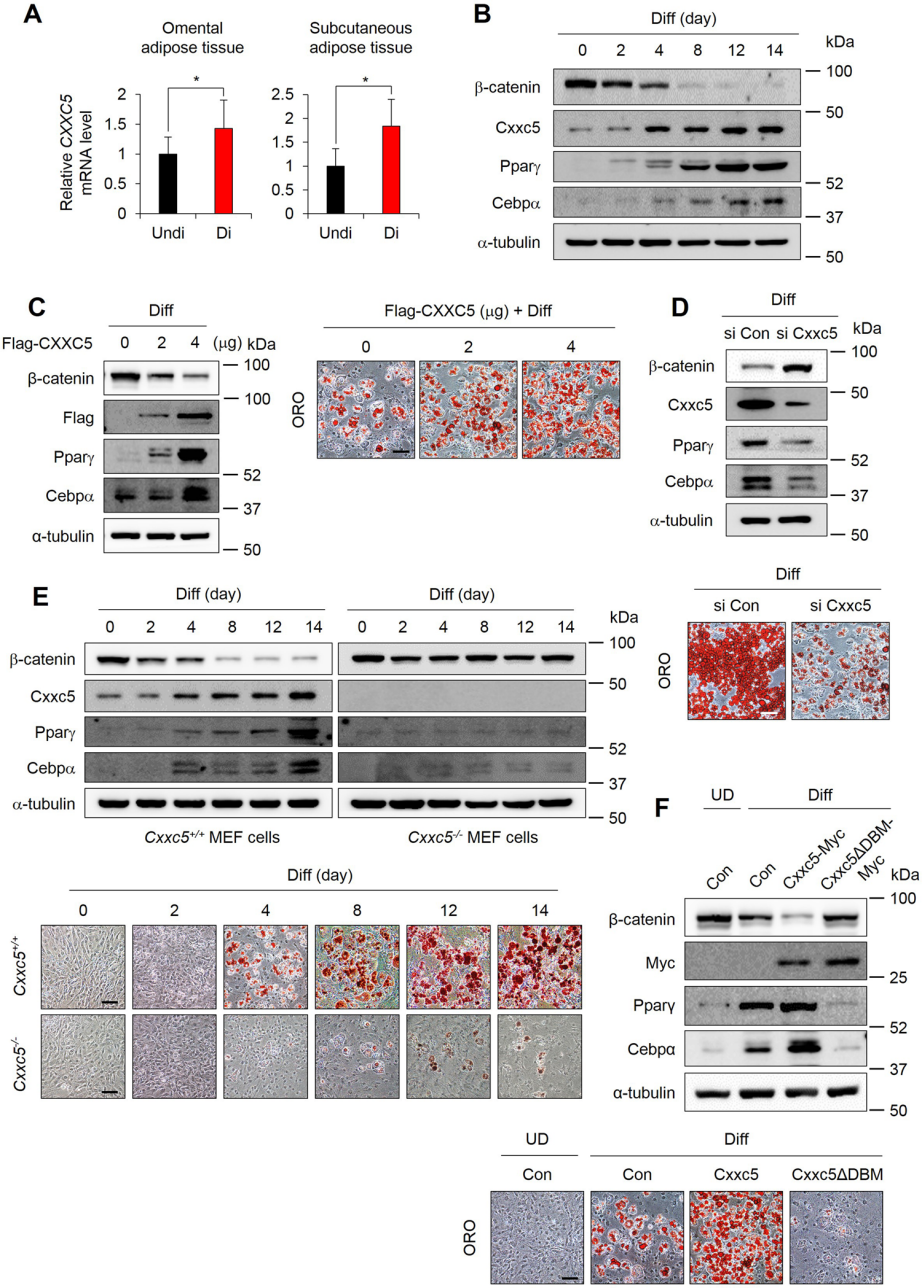
Inhibition of CXXC5-Dvl attenuates adipogenic differentiation in 3T3-L1 preadipocytes. (**A**) expression level of CXXC5 in undifferentiated and differentiated adipocytes of omental and subcutaneous fat pad from patients with obesity (GSE1657, *n* = 3 per group). (**B**) 3T3-L1 preadipocytes were time-dependently differentiated upto 14 days. (**C**) 3T3-L1 preadipocytes were transfected with pcDNA3.0-Cxxc5-Flag at a concentration of 0, 2, 4 µg then differentiated for 7 days. (**D**) 3T3-L1 preadipocytes were transfected with Cxxc5 siRNA and control siRNA then differentiated for 14 days. (**E**) *Cxxc5*^+*/*+^ and *Cxxc5*^*−/−*^ MEFs were time-dependently differentiated upto 14 days. (**F**) 3T3-L1 preadipocytes were transfected with pcDNA3.1-Cxxc5-Myc and pcDNA3.1-Cxxc5ΔDBM-Myc together with corresponding control plasmid then differentiated for 7 days. Scale bar, 100 µm. Results are expressed as mean ± SD., *P < 0.05, versus undifferentiated group. *MEFs* mouse embryonic fibroblasts. *UD* undifferentiated adipocytes, *Diff* differentiated adipocytes.

To identify the relationship between CXXC5 and Wnt/β-catenin signaling in adipocyte differentiation, we checked Cxxc5 and β-catenin levels in adipocytes. The CXXC5 mRNA levels were higher in differentiated adipocytes than in preadipocytes of human omental and subcutaneous adipose tissues (Fig. 1A). The β-catenin levels were gradually reduced with the increment of Cxxc5 during the adipocyte differentiation of 3T3-L1 preadipocytes as monitored by incrementing Pparγ and Cebpα (Fig. 1B). The role of Cxxc5, a negative feedback regulator of the Wnt/β-catenin pathway, in the adipocyte differentiation was confirmed by increment and decrement of both Pparγ and Cebpα through overexpression and siRNA-mediated knockdown of Cxxc5, respectively (Fig. 1C,D). The role of CXXC5 in adipogenic differentiation was further confirmed by suppression of adipocyte differentiation and Pparγ and Cebpα markers with the reduction of lipid droplets in the MEFs isolated from *Cxxc5*^*−/−*^ mice (Fig. 1E). CXXC5 contains a C-terminal Dvl-binding motif (DBM) that is essential for CXXC5 function as a negative regulator of the Wnt/β-catenin pathway^20^. The adipogenic differentiation of 3T3-L1 cells accompanying the increment of Pparγ and Cebpα by overexpression of Cxxc5 was abolished with the restoration of Wnt/β-catenin signaling by overexpression of the Cxxc5ΔDBM (DBM-motif deleted Cxxc5) (Fig. 1F). The role of Cxxc5 in the adipocyte differentiation and important role of its interaction with Dvl were further confirmed by induction of both Pparγ and Cebpα and the deregulation of β-catenin. Cxxc5 level was reduced by treatment of KY19334, a compound that interferes with the Cxxc5-Dvl PPI (Supplemental Fig. 1A,B). KY19334 dose-dependently inhibited the adipogenic differentiation of 3T3-L1 preadipocytes via the Wnt/β-catenin pathway (Supplemental Fig. 1A,B). Overall, CXXC5 plays a role in adipogenic differentiation via binding Dvl and subsequent suppression of the Wnt/β-catenin signaling.

### The HFD-induced obesity and insulin resistance were suppressed by small molecule-mediated blockade of Cxxc5 function

**Figure 2:**
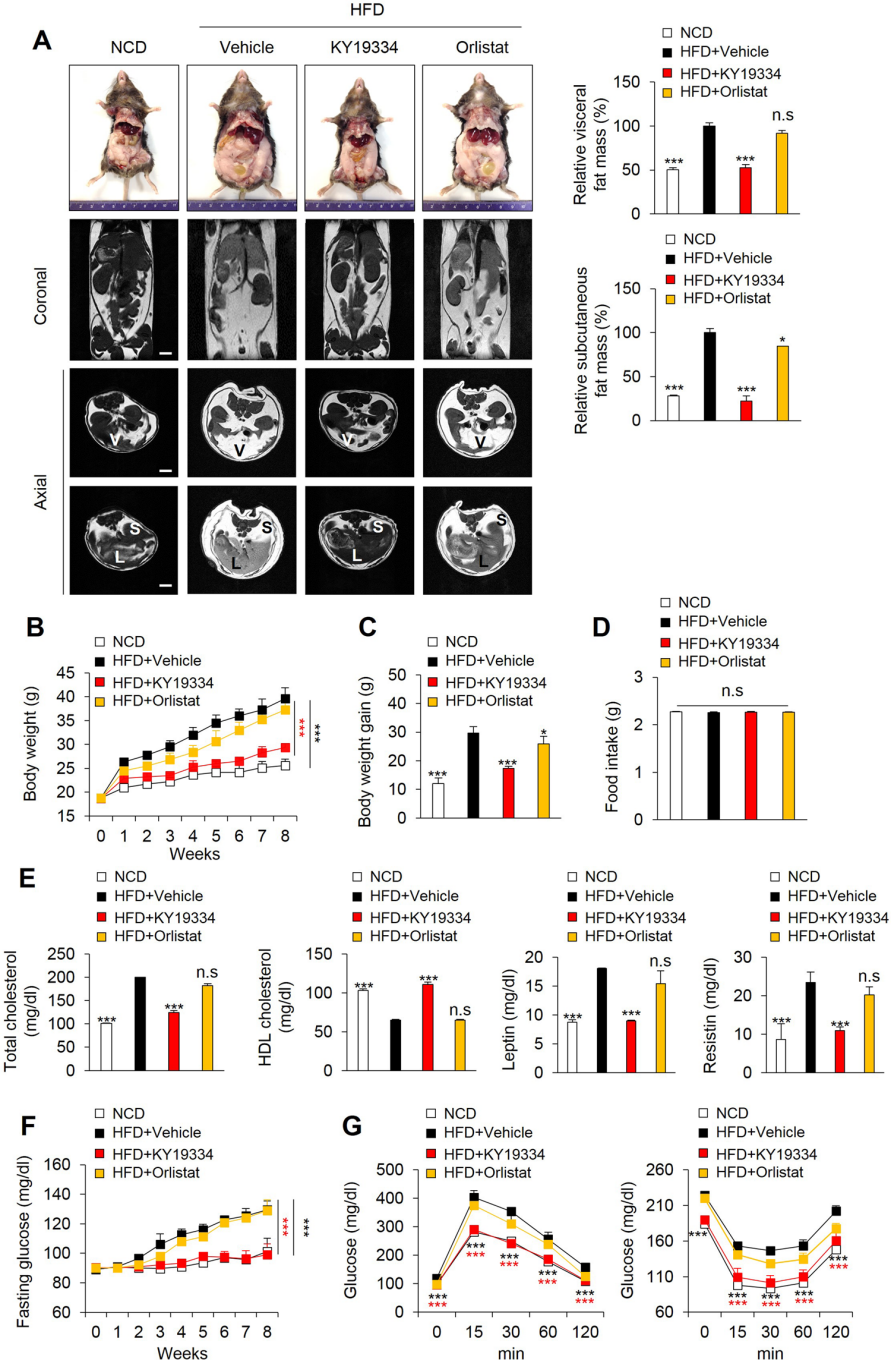
KY19334 treatment prevents diet-induced obesity and insulin resistance. C57BL/6 mice fed the NCD or HFD were orally administered with KY19334 or Orlistat concentration at 25 mg/kg/day for 8 weeks (*n* = 6 per group). (**A**) Representative photographs (upper panel) and MR images of mice (lower panel). Visceral and subcutaneous fat were quantified (**B**) Body weight changes. (**C**) Body weight gain (**D**) Daily food intake during all study weeks (mean ± SD., n.s indicates non-significance with HFD-fed vehicle group). (**E**) Plasma concentration of total cholesterol, HDL-cholesterol, leptin, and resistin in the overnight fasted state. (**F**) Fasting glucose. (**G**) Glucose and insulin tolerance test. Scale bar, 100 µm. Results are expressed as mean ± SD., *n* = 3 per group, *P < 0.05, ***P < 0.001, n.s indicates non-significance with HFD-fed vehicle group. *NCD* normal chow diet, *HFD* high-fat diet.

To investigate the anti-obesity effects of KY19334, it was administered daily for 8 weeks on HFD-fed mice and compared with orlistat, which is a potent gastrointestinal lipase inhibitor^21^. Mice administrated KY19334 was reduced overall fat mass (Fig. 2A and Supplemental Figs. 2, 3A–C), with a significant reduction in both visceral and subcutaneous fats compared with those of the orlistat-treated HFD mice at the same dose (Fig. 2A). The HFD-fed mice administered KY19334 showed less body fat mass as accounted for the lower body weight gain compared to that of the HFD mice applied orlistat without significant changes in food intake (Fig. 2B–D and Supplemental Fig. 3D). Consistent with the reduction of obesity, treatment of KY19334, but not orlistat, specifically improved their metabolic parameters including total cholesterol, HDL-cholesterol, leptin, and resistin as well as the blood glucose level and insulin sensitivity (Fig. 2E–G and Supplemental Fig. 3E–G). Overall, KY19334, the CXXC5-Dvl PPI inhibitor, improved metabolic parameters with the anti-obesity effect.

### KY19334 reduces adiposity and inflammation in the epiWAT of HFD-fed mice

**Figure 3:**
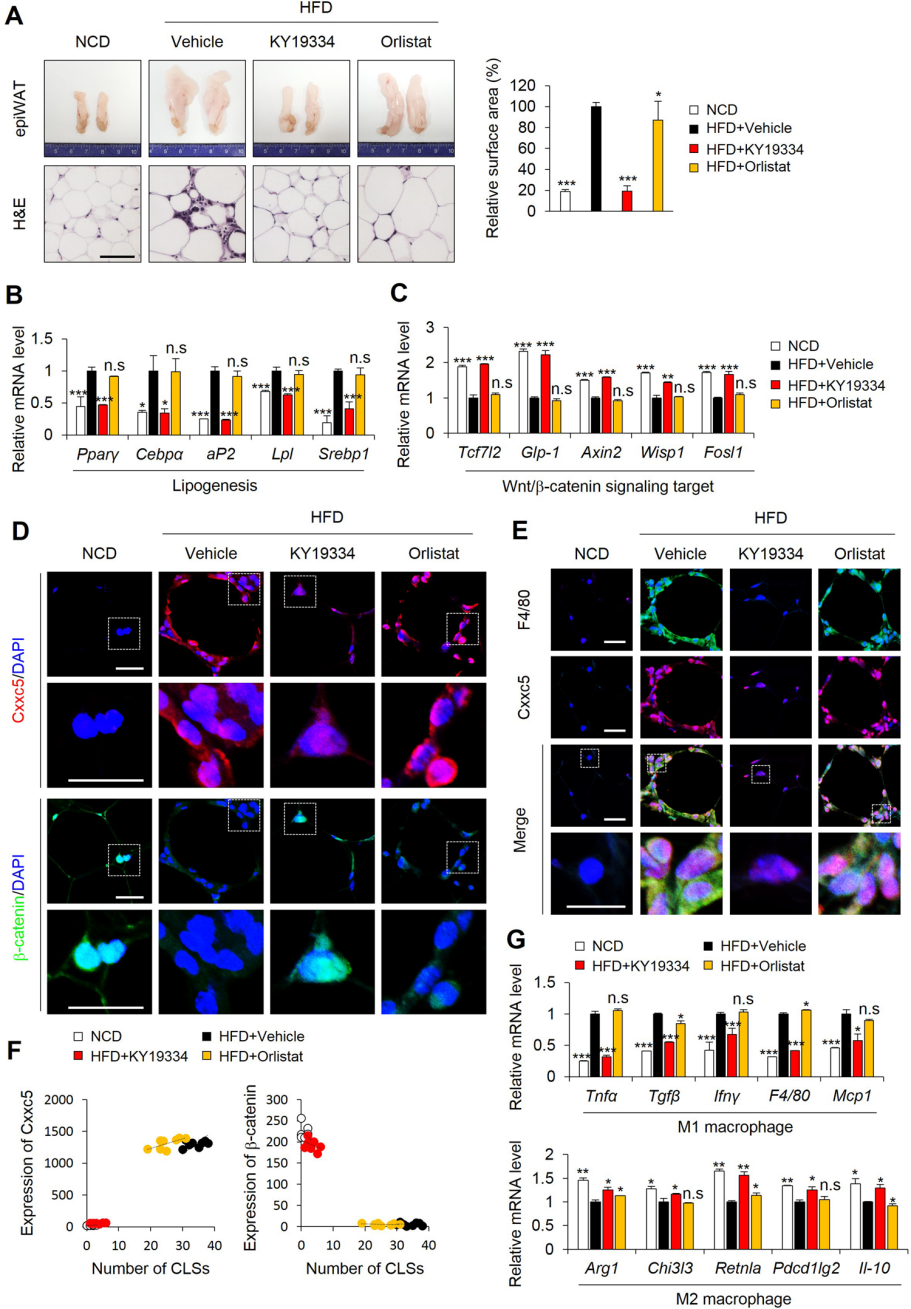
KY19334 treatment reduces adipogenesis and inflammation in epiWAT. C57BL/6 mice fed the NCD or HFD were administered as described in the legend of Fig. 2. (**A**) Representative images of epiWAT and H&E staining. Quantification analyses of adipocyte cell size of epiWAT. (**B**,**C**) Relative expression levels of marker genes for lipogenesis (**B**) and Wnt/β-catenin signaling target (**C**) (*n* = 3 per group). (**D**) Representative IHC images for Cxxc5 and β-catenin in the epiWAT. Quantitative mean intensity value of IHC staining of Cxxc5 and β-catenin. (**E**) Representative IHC images for F4/80 and Cxxc5 in the epiWAT. (**F**) The correlation of Cxxc5 and β-catenin expression with the number of CLSs. (**G**) Relative expression levels of marker genes for M1 and M2 macrophage. Scale bar, 100 µm. Results are expressed as mean ± SD., *n* = 3 per group, *P < 0.05, **P < 0.01, ***P < 0.001, n.s indicates non-significance with HFD-fed vehicle group. *NCD* normal chow diet, *HFD* high-fat diet, *epiWAT* epididymal white adipose tissue, *CLSs* crown-like structures.

The decrease in obesity could be attributed to a reduction in adipocyte size due to a reduction in the inflammatory response in the epididymal white adipose tissue (epiWAT)^22,23^. Treatment of KY19334 significantly reduced the fat mass and size of adipocytes with the lower expression levels of lipogenesis marker genes in epiWAT compared with those of orlistat-treated mice (Fig. 3A,B). The Wnt/β-catenin signaling target genes were up-regulated with the elevation of nuclear β-catenin in the epiWAT of KY19334-treated mice (Fig. 3C,D). The decrement of nuclear β-catenin and the induction of Cxxc5 protein in F4/80 + crown-like structures (CLSs) was observed in the epiWAT of vehicle- and orlistat-treated HFD mice (Fig. 3D–F). The F4/80 + CLSs indicating inflammation was significantly reduced with the abolishment of Cxxc5 in the epiWAT of KY19334-treated mice (Fig. 3D–F). The mRNA expression levels of M1 macrophage were decreased with the increment of M2 macrophage marker genes in the epiWAT of KY19334-treated mice (Fig. 3G). Overall, KY19334 suppressed inflammation in epiWAT with a reduction of adiposity in HFD-fed mice.

### KY19334 induces a browning phenotype with the adiposity reduction in scWAT of HFD-fed mice

**Figure 4:**
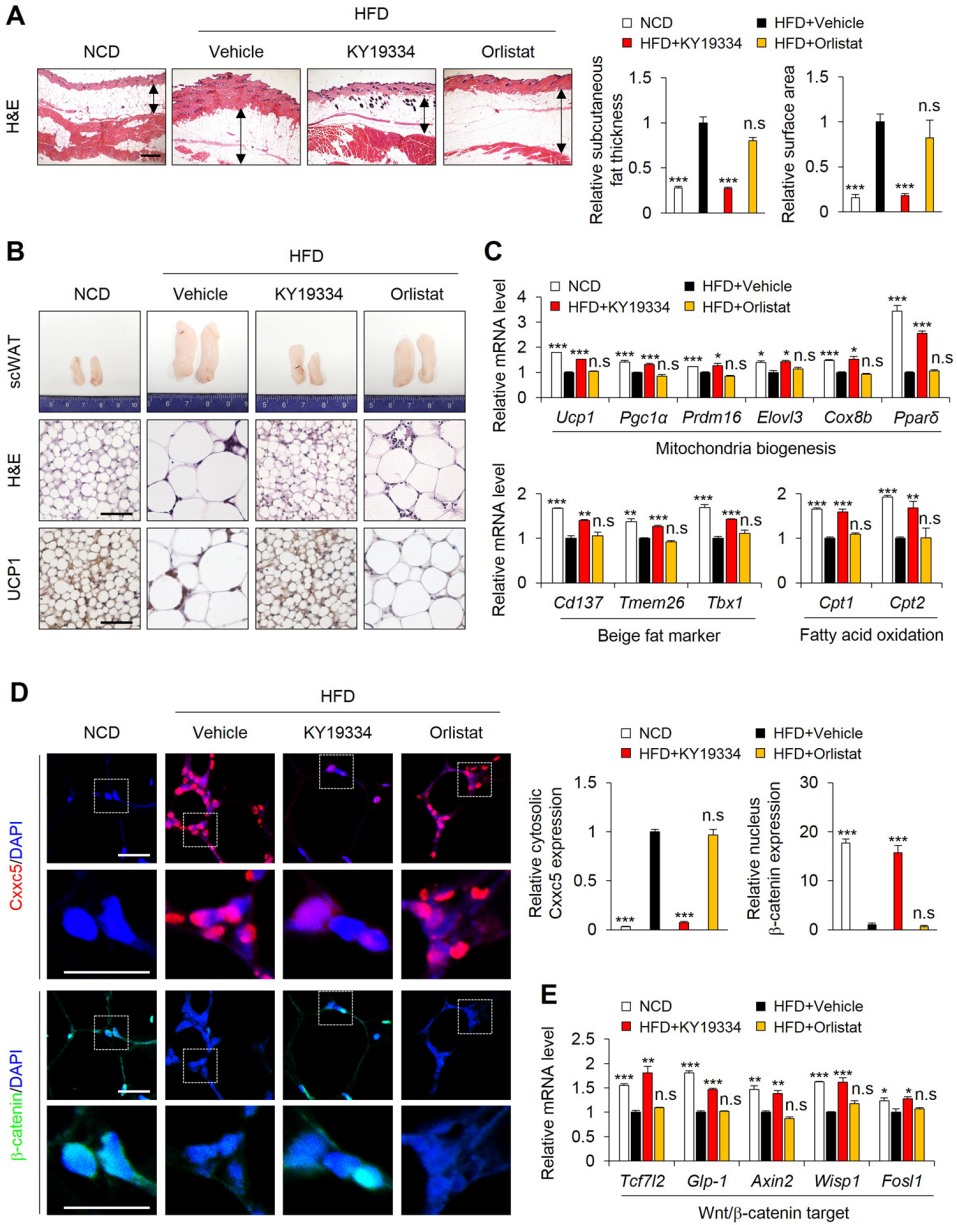
KY19334 treatment reduces adipogenesis and promotes adipose tissue browning in scWAT. C57BL/6 mice fed the NCD or HFD were administered as described in the legend of Fig. 2. (**A**) Representative images of H&E staining of scWAT. Scale bar, 100 µm. Quantitative analyses of subcutaneous fat thickness and adipocyte cell size. (**B**) Representative images of scWAT, H&E staining, and Ucp1 immunohistochemistry of scWAT. (**C**) Relative expression levels of marker genes for mitochondria biogenesis, beige fat and fatty acid oxidation. (**D**) Representative IHC images for Cxxc5 and β-catenin in the scWAT (left panel) and quantitative analyses of IHC staining for CXXC5 and β-catenin (right panel). (**E**) Relative expression levels of marker genes for Wnt/β-catenin target. Scale bar, 100 µm. Results are expressed as mean ± SD., *n* = 3 per group, *P < 0.05, **P < 0.01, ***P < 0.001, n.s indicates non-significance with HFD-fed vehicle group. *NCD* normal chow diet, *HFD* high-fat diet, *scWAT* subcutaneous white adipose tissue, *Ucp1* uncoupling protein 1.

The adipose tissue browning, which protects against metabolic dysfunction, is more susceptible to subcutaneous white adipose tissue (scWAT) than epiWAT^24^. To further characterize the adiposity suppression by KY19334, the KY19334 effect on browning scWAT was investigated in HFD-fed mice. The fat thickness between the skin and muscle layers was significantly decreased by KY19334 treatment compared with those revealed by vehicle- and orlistat-treated HFD mice (Fig. 4A). In addition, both mass and size of fats were substantially reduced in scWAT of KY19334-treated mice (Fig. 4A,B). The critical increment of brown fat-like depots was shown by an increment of the expression of Ucp1 accompanying increment of the key mitochondria biogenesis, beige-fat, and fatty acid oxidation markers in scWAT of KY19334-treated HFD mice (Fig. 4B,C). Meanwhile, KY19334 treatment reduced Cxxc5 with F4/80 + CLSs, overlapping in the scWAT compared with the vehicle- and orlistat-treated mice (Supplemental Fig. 4A,B). The mRNA expression levels of M1 and M2 macrophage marker genes were decreased and increased, respectively, in the scWAT of KY19334-treated mice (Supplemental Fig. 4C). Compared to the vehicle- and orlistat-treated mice, the expression of Cxxc5 was mostly abolished in scWAT and weakly remained in the nucleus with an increment of nuclear β-catenin, especially in nuclei of the scWAT of KY19334-treated HFD mice (Fig. 4D). In addition, the Wnt/β-catenin signaling target genes were up-regulated in the scWAT of KY19334-treated mice (Fig. 4E). Therefore, KY19334 increases energy metabolism with the correlative reduction of adiposity in scWAT of HFD-fed mice.

### KY19334 reduces hepatic steatosis and improves hepatic glucose homeostasis

**Figure 5:**
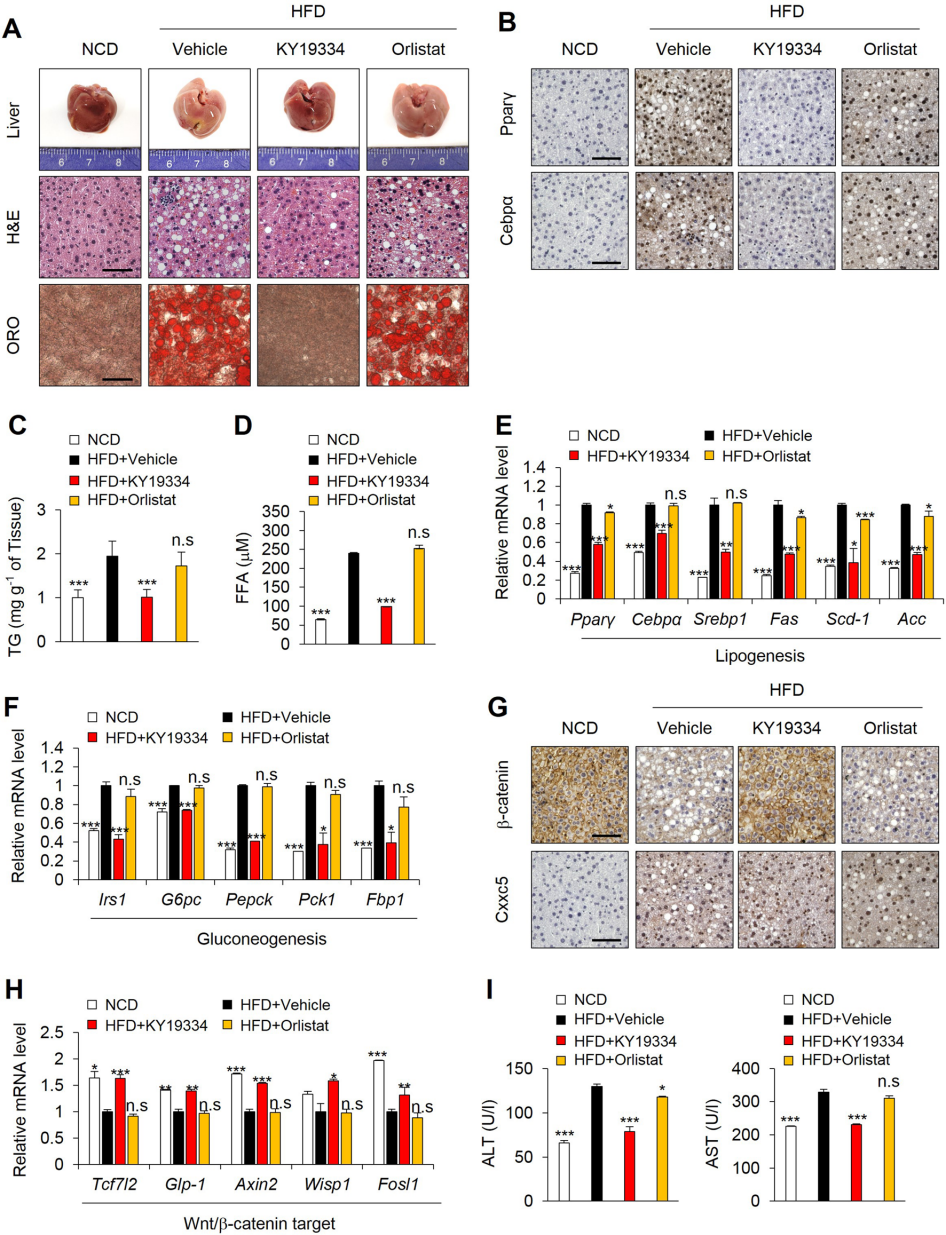
KY19334 treatment prevents hepatic steatosis and improves glucose homeostasis. C57BL/6 mice fed the NCD or HFD were administered as described in the legend of Fig. 2. (**A**) Representative images of liver, H&E staining, and Oil Red O staining. (**B**) Representative images of Pparγ and Cebpα immunohistochemistry of liver. (**C**) TG concentration in liver tissues. (**D**) Plasma concentration of FFA. (**E**,**F**) Relative expression levels of marker genes for lipogenesis (**E**) and gluconeogenesis (**F**) (*n* = 3 per group). (**G**) Representative images of β-catenin and Cxxc5 immunohistochemistry of liver. (**H**) Relative expression levels of marker genes for Wnt/β-catenin target. (**I**) Plasma concentration of ALT and AST. Scale bar, 100 µm. Results are expressed as mean ± SD., *n* = 3 per group, *P < 0.05, **P < 0.01, ***P < 0.001, n.s indicates non-significance with HFD-fed vehicle group. *NCD* normal chow diet, *HFD* high-fat diet, *TG* triglyceride, *FFA* free fatty acid, *ALT* alanine transaminase, *AST* aspartate transaminase.

The liver tissues of KY19334-treated mice did not form lipid droplets with a reduction in Pparγ and Cebpα-positive cells compared with those of vehicle- and orlistat-treated mice on HFD (Fig. 5A,B). The reduction of hyperlipidemia and hyperglycemia by KY19334 treatment was confirmed by hepatic triglyceride (TG) and free fatty acid (FFA) levels (Fig. 5C,D). Consistently with the improvement of hepatic glucose homeostasis by KY19334 treatment, the expression of lipogenesis and gluconeogenesis genes were suppressed in the liver tissue cells of KY19334-treated mice (Fig. 5E,F). In addition, β-catenin was significantly increased with a decrement of Cxxc5 in KY19334-treated mice with the induction of Wnt/β-catenin signaling target genes (Fig. 5G,H). The HFD-induced increment of factors in serum indicating liver damage, such as ALT and AST, were decreased in KY19334-treated HFD mice compared to those of vehicle- and orlistat-treated mice (Fig. 5I), indicating that KY19334 improves hepatic glucose homeostasis.

## Discussion

The current clinically applicable pharmacological agents for anti-obesity, including orlistat, lorcaserin, liraglutide, and phentermine/topiramate, have been prescribed for weight loss with the mechanisms of reduction of calorie absorption or appetite. However, low efficacy and safety issues have resulted in their limitations in developing pharmaceutical agents^5,25^. Growing pieces of evidence indicate that substantial early weight loss by targeting the metabolic tissues such as adipose tissue, liver, and skeletal muscle have importance on efficacy and sustainability of anti-obesity^26^.

In the present study, we provided an approach for suppressing early adipogenic differentiation during the development of obesity. By illustrating the role of Cxxc5 in inhibiting the Wnt/β-catenin pathway related to adipogenic differentiation and a small molecule-mediated interference of the Cxxc5 function up-regulating Wnt/β-catenin signaling is suggested as a new potential anti-obesity target.

The pathological significance of the CXXC5 function was indicated by suppression of Wnt/β-catenin signaling with overexpression of CXXC5 in the adipocyte tissues of patients with obesity as well as the early adipogenic differentiation of preadipocytes. The role of Cxxc5, which suppress Wnt/β-catenin signaling in the adipogenesis was confirmed by the phenotypes of the *Cxxc5*^*−/−*^ MEFs as well as the knockdown of *Cxxc5* in adipogenic differentiation in vitro.

The anti-obesity effects of KY19334 could be acquired by suppression of early adipogenic differentiation, the initial event of obesity, accomplished by controlling the function of CXXC5 by the interference of the Cxxc5-Dvl PPI. Administration of KY19334, which restores the suppressed Wnt/β-catenin pathway in the HFD-induced obese mouse, showed anti-obesity effects with the suppression of insulin resistance, inflammation, and hepatic steatosis as well as the induction of adipose tissue browning. The KY19334-mediated suppression of hypertrophy of adipose tissues accompanying improvement of various metabolic factors during adipogenesis in the HFD-fed mice did not or marginally occur by administration of the orlistat, a lipase inhinitor^27^. Fundamentally different with orlistat, KY19334 suppress inflammation with the improvement of obesity by the Wisp1-mediated suppression of expression of the Pparγ and Cebpα^9^. Moreover, many other factors which are directly induced by Wnt/β-catenin signaling such as TCF7L2, GLP-1, PPARγ, FGF19, and FGF21 are also attributed to the anti-obesity accompanying suppression of inflammation^28–30^, hepatic steatosis^30,31^, and activation of browning of WAT^32^, resulting in improvement of the multiple metabolic parameters. Furthermore, ectopic expression of UCP1 in scWAT from KY19334-treated mice was linked to protecting diet-induced obesity by increased fatty acid oxidation of scWAT.

The role of CXXC5 in obesity was correlated with high induction of the inflammation markers and cytosolic accumulation of CXXC5 was inversely correlated with β-catenin in the metabolic tissues of the HFD-induced obese mice.

Overall, CXXC5 overexpression plays a role as a major driver in the adipogenic differentiation the obesity. An approach for restoring the suppressed Wnt/β-catenin signaling via blockade of CXXC5-Dvl PPI offer a novel approach for anti-obesity with the improvement of metabolic parameters on HFD-mediated over nutrition. In addition, this approach activating the Wnt/β-catenin signaling, not by direct activation, but by release of the suppressed Wnt/β-catenin signaling by blockade of a negative feedback mechanism by CXXC5 could provide a safe anti-obesity approach effect which is a major huddle in the development of anti-obesity drugs and targeting the Wnt/β-catenin pathway. The small molecule approach interfering CXXC5 function provides a potential treatment of overall metabolic abnormalities as well as obesity involving over nutrition related to the HFD.

## Methods

### Animals

Twenty-four male C57BL/6 mice (5-week-old) were purchased from KOATECH (Seoul, Korea). The mice were housed in a pathogen-free facility at Yonsei University (Seoul, Korea). After a 1-week acclimation period with a commercial diet and tap water, mice were weight matched and divided into four different dietary groups (*n* = 6 each group): NCD or HFD consisting of 60% calories from fat (Research Diet, D12492, Research Diets, Inc., New Brunswick, NJ, USA). HFD-fed mice were orally administered with KY19334 (25 mg/kg) or orlistat (25 mg/kg) for 8 weeks. Body weight and food intake were measured once a week during the study. Assessment of fasting glucose levels, glucose tolerance test (GTT) and insulin tolerance test (ITT) were described in a previous study^17^. All mice were maintained under temperature and light-controlled (standard 12 h light/dark cycle) conditions and provided with food and water ad libitum. All animal experiments were conducted in compliance with the guidelines which were reviewed and approved by the Institutional Review Board of Severance Hospital, Yonsei University College of Medicine (09-013). The study was carried out in compliance with the ARRIVE guidelines.

### MRI (Magnetic resonance imaging)

Bruker 7-Tesla small animal magnetic resonance imaging system with a 12-cm radiofrequency coil was used to visualize and non-invasively quantify various fat deposits. 3D rapid acquisition with relaxation enhancement (RARE) imaging sequence was used for high-contrast fat imaging: TE 5.9, TR 200, RARE factor 8, flip angle 60, matrix 256 × 128 × 128, 7 × 3 × 3 cm to 9 × 3 × 5 cm (cranial-caudal AP LR). Using this protocol, a 3D-reconstructed image was produced using a maximum intensity projection algorithm with an intensity threshold that shows fat as the brightest signal and disregards signals from other tissues. The total body fat and separated specific fat deposits were calculated using thresholding and voxel count plug-ins from NIH ImageJ software and a VolumeJ plug-in to create three-dimensional fat images. An animal management system in conjunction with the MRI was used to record core, skin, ambient and water-blanket temperature measurements for monitoring during imaging. The core temperature of the animal was maintained during imaging and anesthesia induction using a water blanket maintained at 37 °C.

### Blood chemistry

Total blood of mice was collected by cardiac puncture after fasting. The blood was allowed to clot for 30 min and was then centrifuged for 10 min at 1000×*g* to obtain supernatant to measure metabolic parameters^17^. ELISA assay kits were used to assess serum leptin (R&D system, MOB00B), serum resistin (R&D system, MRSN00), serum FFA (Cayman Chemical, 700310). Serum chemistry variables included total cholesterol, high-density lipoprotein (HDL)-cholesterol, glucose, TG, alanine aminotransferase (ALT), and aspartate transaminase (AST) concentrations. The calibration of serum parameters was performed using the quality control card supplied with the FUJI DRI-CHEM slides whenever slides from a new lot were used.

### Hematoxylin and eosin (H&E) staining

H&E staining was performed as previously described^17^. Dissected tissues were fixed in 4% neutral paraformaldehyde and embedded in paraffin. The paraffin sections were cut at a thickness of 4 µm and subjected to H&E staining. The adipocyte cell size was measured in 20 randomly chosen microscopic areas from 3 independent animals using a Nikon bright-field optical microscope (Nikon TE-2000U). The average adipocyte size and dermal thickness were determined using Image J software.

### Immunohistochemistry (IHC)

IHC staining was performed as previously described^17^. Paraffin sections of 4 µm in size were deparaffinized and rehydrated. For antigen retrieval, the slides were autoclaved in 10 mM sodium citrate buffer (pH 6.0). Sections were blocked in phosphate buffered saline (PBS) containing 10% BSA at 20 °C for 30 min. The sections were incubated overnight at 4 °C with the following dilution of primary antibodies: anti-β-catenin (BD, 610154), anti-CXXC5 (Lab made), and anti-F4/80 (Cell Signaling Technology, sc-377009). The slides were washed with PBS, incubated with Alexa Fluor 488- (Invitrogen, A-11001) or Alexa Fluor 555-conjugated IgG secondary antibody (Invitrogen, A-21428) at 20 °C for 1 h, and counterstained with DAPI (Sigma-Aldrich, D9564). The images were captured using a LSM700 META confocal microscope (Carl Zeiss) after excitation with 405-, 488-, or 543-nm laser lines.

To block endogenous peroxidase activity before peroxidase IHC analysis, tissues were incubated with 1% H_2_O_2_ (Samchun Chemicals) for 10 min, then blocked in PBS containing 10% BSA at 20 °C for 30 min Sections were incubated with primary antibody overnight at 4 °C with the following dilution of primary antibodies: anti-uncoupling protein 1 (UCP1; Abcam, ab10983), anti-β-catenin (BD, 610154), anti-CXXC5 (Lab made), anti-PPARγ (Santa Cruz, SC-271392), and anti-C/EBPα (Cell Signaling Technology, 2295). Then, sections were incubated with biotinylated anti-rabbit (Dako, BA-1000) or anti-mouse (Dako, BA-9200) secondary antibody for 1 h at 20 °C. The samples were stained with 3, 3′-diaminobenzidine (DAB; Dako, SK-4100) for 3–7 min and counter stained with Mayer’s hematoxylin (Muto). All incubations were conducted in humid chambers. Signals were analyzed using a bright field microscope (Nikon TE-2000U).

### TG assay

Liver tissues were incubated on ice in 100 µl saline solution (2 M NaCl, 2 mM EDTA, 50 mM sodium phosphate, and pH 7.4). Cells and tissues suspensions were assayed for TG content using a TG assay kit (Cayman Chemical, CBL-STA-396).

### Quantitative real-time polymerase chain reaction (PCR)

Real-time PCR was performed as previously described^17^. Briefly, total RNA was extracted from ground tissue powder using TRIzol reagent (Invitrogen) according to the manufacturer’s instructions. Reverse transcription was performed with M-MLV reverse transcriptase (Invitrogen) using 2 µg of total RNA. Synthesized cDNA was diluted as a concentration of 100 ng/µl. Quantitative PCR analyses were performed in the Rotor-gene Q real-time PCR cycler (Qiagen) using SYBR green reagent (Qiagen) with conditions of 95 °C for 10 min followed by 40 cycles at 95 °C for 5 s and 60 °C for 15 s. Relative quantification of mRNA levels was estimated using the comparative Ct method (∆∆Ct). All mRNA values were normalized with respect to *GAPDH*. The primer sequences are listed in Table S1.

### Isolation of MEF cells

Primary *Cxxc5*^+*/*+^ and *Cxxc5*^*−/−*^ MEFs were prepared from E13.5-E14.5 embryos. The embryos were cut into small pieces and enzymatically digested using 0.5% Trypsin/EDTA (Gibco). Post-digestion, the tissue was resuspended in DMEM and pipetted through a 40 µM filter (BD) to obtain single cells. MEFs were expended and frozen.

### Cell culture, transfection, and adipocyte differentiation

3T3-L1 preadipocytes were maintained in DMEM (Gibco) containing 10% (vol/vol) heat-inactivated calf serum (CS; Gibco), 100 mg/ml penicillin (Gibco), and 100 mg/ml streptomycin (Gibco). *Cxxc5*^+*/*+^ and *Cxxc5*^*−/−*^ MEFs were maintained in DMEM containing 10% (vol/vol) heat-inactivated fetal bovine serum (FBS; Gibco), 100 mg/ml penicillin/streptomycin, and 2 mM L-glutamin (Gibco). For transient transfection, the cells were grown for 1 day, transfected with the required plasmid or siRNA using the Attractene transfection reagent (Qiagen) in Opti-MEM. After 16 h, the medium was changed to DMEM supplemented with 10% CS, and cells were induced to differentiate in DMEM containing 10% FBS supplemented with MDI (520 µM methylisobutylxanthine, 1 µM dexamethasone and 167 nM insulin) with or without KY19334. After 2 days, the medium was replaced with DMEM containing 10% FBS and 167 nM insulin with or without KY19334. The medium was changed with fresh identical medium every 2 days up to day 14 post-induction. Undifferentiated control groups were maintained in DMEM supplemented with 10% CS, 100 mg/ml penicillin, and 100 mg/ml streptomycin. The cells were incubated in 5% (vol/vol) CO_2_ at 37 °C.

### Western blotting

Western blot analysis was performed as previously described^17^. Cells were lysed using radio-immunoprecipitation assay (RIPA) buffer (150 mM NaCl, 10 mM Tris, pH 7.2, 0.1% SDS, 1.0% Triton X-100, 1% sodium deoxycholate, and 5 mM EDTA). Samples were separated on 12% SDS polyacrylamide gels and transferred onto PROTRAN nitrocellulose membranes (Shleicher and Schuell Co.). After blocking with PBS containing 5% nonfat dry skim milk and 0.07% (vol/vol) Tween 20, the membranes were incubated with antibody specific for β-catenin (1:1000, sc-7963, Santa Cruz Biotechnology, Inc.), CXXC5 (1:500, Lab made), PPARγ (1:1000, ab19481, Abcam), or C/EBPα (1:1000, 2295, Cell Signaling Technology) at 4 °C overnight. Membranes were then incubated with horseradish peroxidase-conjugated anti-rabbit (1:1000, 170-6515, Bio-Rad) or anti-mouse (1:1000, 14790, Cell Signaling Technology) IgG secondary antibody. Protein bands were visualized with enhanced chemiluminescence (GE Healthcare) using a luminescent image analyzer, LAS-3000.

### Oil Red O staining

Oil red O staining was performed as previously described^17^. Liver tissues and cells were washed with PBS, 70% isopropanol (Duksan Pure Chemicals), and stained with Oil Red O solution (Sigma-Aldrich) at 20 °C overnight. Samples were washed thoroughly with distilled water. Tissues were counterstained with Mayer’s hematoxylin. Images of the Oil Red O staining were visualized with a bright field microscope (Nikon TE-2000U). For the quantification of lipid contents, the Oil Red O was eluted by the addition of 500 µl isopropanol containing 4% nonidet P-40 to each well and the absorbance was measured spectrophotometrically at 590 nm.

### Bioinformatic data analyses

The gene expression profile results (Gene ID: 51523) were deposited in NCBI’s GEO database (https://www.ncbi.nlm.nih.gov/geo/) and either of the two human omental (undifferentiated: GSM30651, GSM30652, GSM30653 *vs*. differentiated: GSM30660, GSM30661, GSM30662) and subcutaneous adipose tissue (undifferentiated: GSM30645, GSM30646, GSM30647 *vs*. differentiated: GSM30654, GSM30655, GSM30656) transcriptome datasets are accessible through GEO accession number GSE1657.

### Statistical analysis

Data are presented as means ± standard deviation (SD). Statistical analyses were performed using unpaired two-tailed Student’s *t*-test. Asterisks denote statistically significant differences (*P < 0.05; **P < 0.01; ***P < 0.001).

## Supplementary Information

Supplementary Information.

## Data Availability

Raw and processed data from the next-generation RNA sequencing of samples were deposited in the NCBI Gene Expression Omnibus (GEO) under accession numbers HG-U133B. All data that support the findings of this study are available from the corresponding authors upon request.
