# Supplementary material for: Blockade of CXXC5-dishevelled interaction inhibits adipogenic differentiation, obesity, and insulin resistance in mice
Source: Sci Rep. 2022 Nov 30;12:20669. doi: 10.1038/s41598-022-25315-x (PMC9712602; doi:10.1038/s41598-022-25315-x)
Supplement: Supplementary file 1 — Supplementary Information. [file 41598_2022_25315_MOESM1_ESM.docx]

**Supplementary information**

**Blockade of CXXC5-Dishevelled interaction inhibits adipogenic differentiation, obesity, and insulin resistance**

**Seol Hwa Seo^1^, Dasung Lee^1^, Soung-Hoon Lee^2^, Kang-Yell Choi^1,2^**^🖂^

^1^ Department of Biotechnology, College of Life Science and Biotechnology, Yonsei University, Seoul, 03722, Republic of Korea. ^2^ CK Regeon Inc, Seoul, 03722, Republic of Korea. ^🖂^email: kychoi@yonsei.ac.kr

**Immunoprecipitation assay.** Immunoprecipitation assay was performed as previously described^1^. To monitor the protein-protein interactions, 1 mg of WCLs were incubated with anti-Myc and protein A agarose beads (GenDEPOT) at 4°C for 16 h, and the beads were then washed three times in RIPA buffer. The resulting immune complexes were resolved by SDS-PAGE, and the membranes were incubated with antibody specific for Dvl (1:1,000, sc-8025, Santa Cruz Biotechnology, Inc.), and Myc (1:1,000, M192-3, MBL) at 4°C overnight. Membranes were then incubated with horseradish peroxidase-conjugated anti-mouse (1:1,000, 14790, Cell Signaling Technology) IgG secondary antibody. Protein bands were visualized with enhanced chemiluminescence (GE Healthcare) using a luminescent image analyzer, LAS-3000.

**Reference**

1. Kim HY, Yoon JY, Yun JH, et al. CXXC5 is a negative-feedback regulator of the Wnt/β-catenin pathway involved in osteoblast differentiation. Cell Death Differ. 2015;22: 912-20.

**
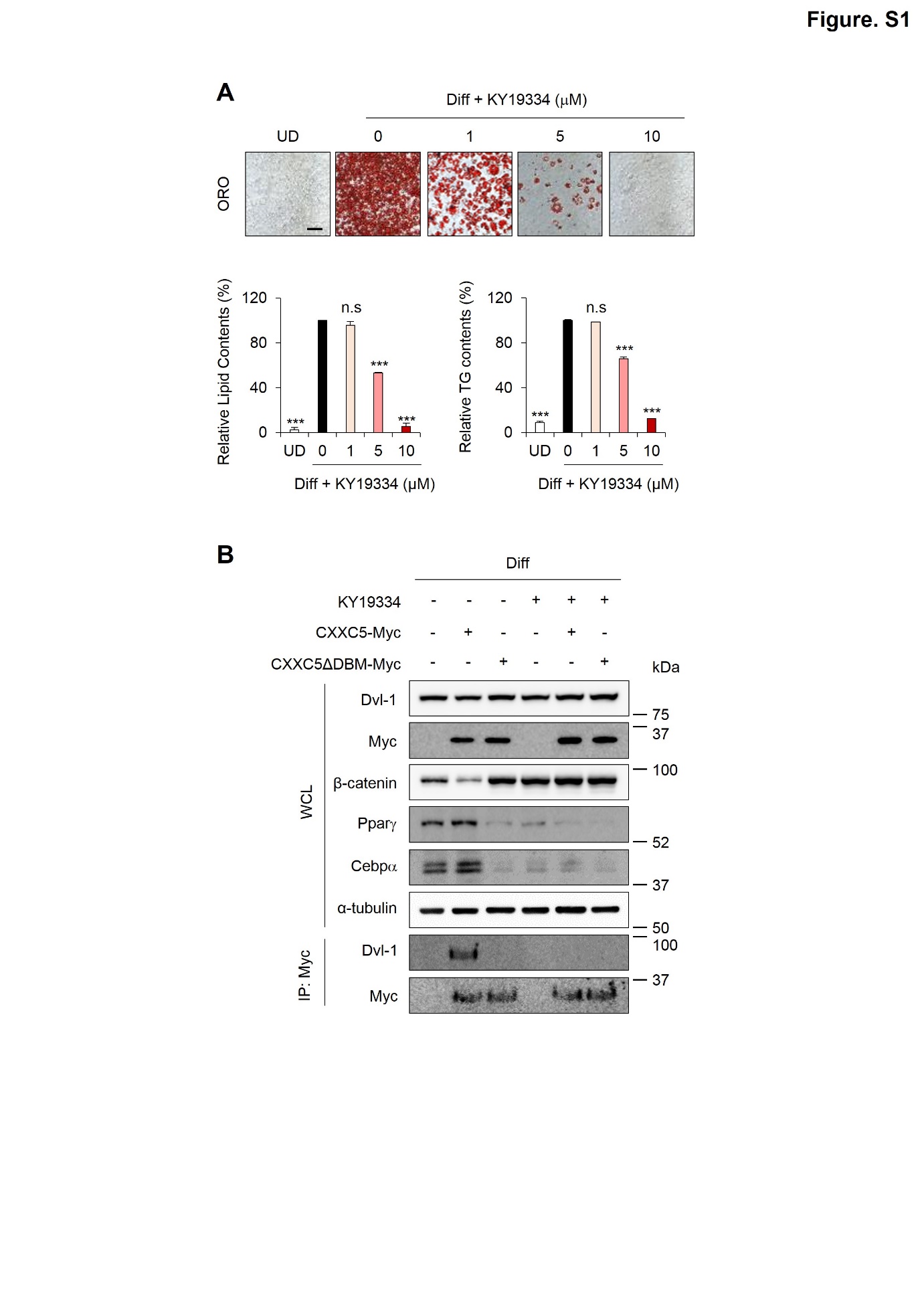
**

**Supplementary Fig. 1. Functional characterization of the effects KY19334 on adipocyte differentiation via inhibition of CXXC5-Dvl.** (**A**) 3T3-L1 preadipocytes were treated with KY19334 and induced adipogenic differentiation. At 14 days of differentiation, intracellular accumulation of lipid was assessed by ORO staining and lipid and triglyceride content were quantified. (**B**) 3T3-L1 preadipocytes transfected with pcDNA3.1, pcDNA3.1-Cxxc5-Myc, and pcDNA3.1-Cxxc5ΔDBM-Myc together were treated with or without 10 µM of KY19334 then differentiated for 7 days. The cells were induced differentiate using MDI and whole cell lysates were subjected to Western blot analyses to detect the protein levels of β-catenin, Cxxc5, Pparγ, and Cebpα or the intracellular accumulation of lipid were observed by ORO staining.


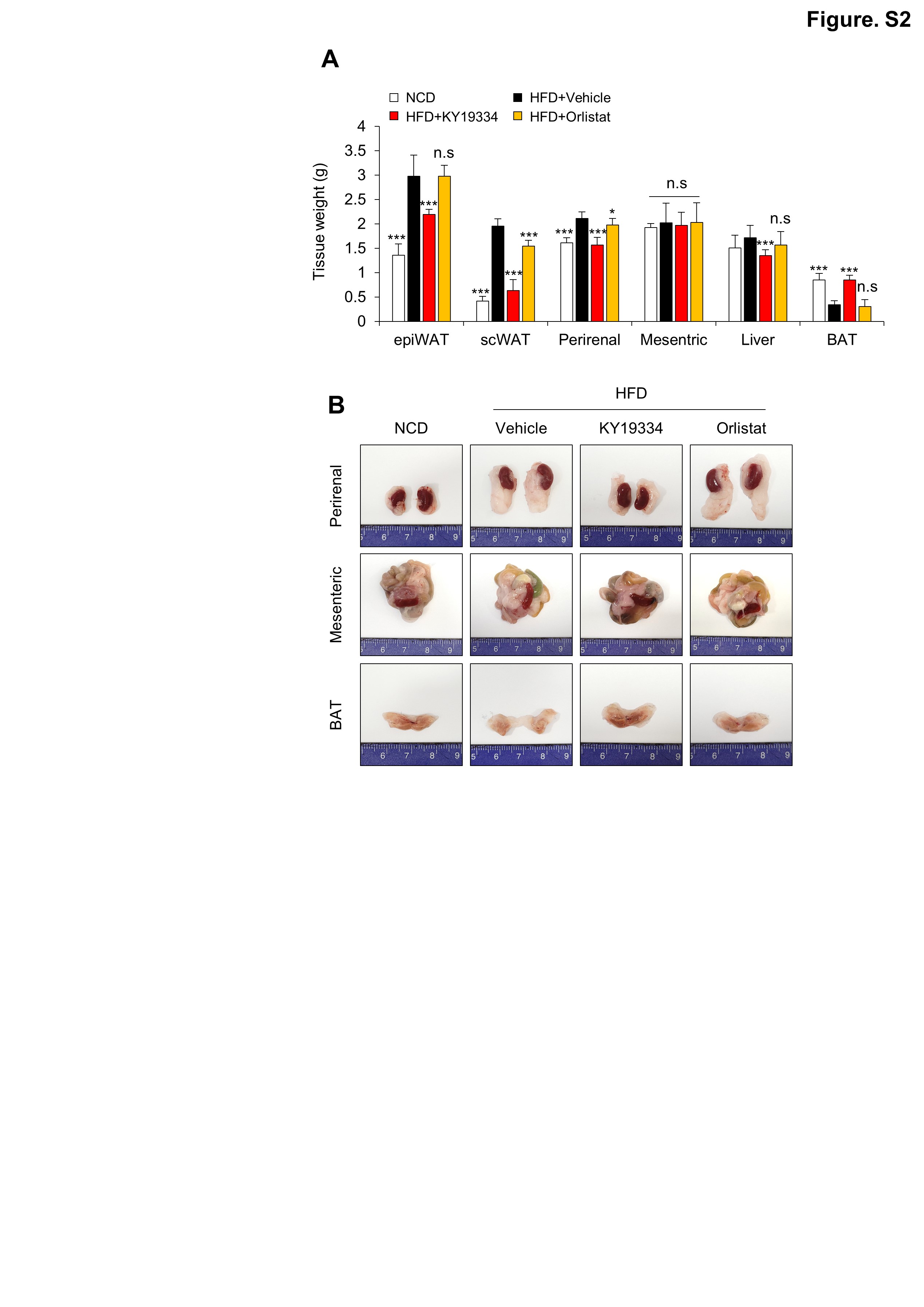


**Supplementary Fig. 2. KY19334 treatment reduces WAT weight and mass.** (**A**,**B**) The data shown were obtained from the same mice that examined in Fig. 2. Wet weight of epiWAT, scWAT, perirenal, mesenteric, liver, and BAT (mean ± SD., **P* < 0.05, ****P* < 0.001, n.s indicates non-significance with HFD-fed vehicle group) (**A**) and representative images of perirenal, mesenteric, and BAT (**B**). Results are expressed as mean ± SD., *n* = 3 per group, **P* < 0.05, ***P* < 0.01, ****P* < 0.001, n.s indicates non-significance with HFD-fed vehicle group.


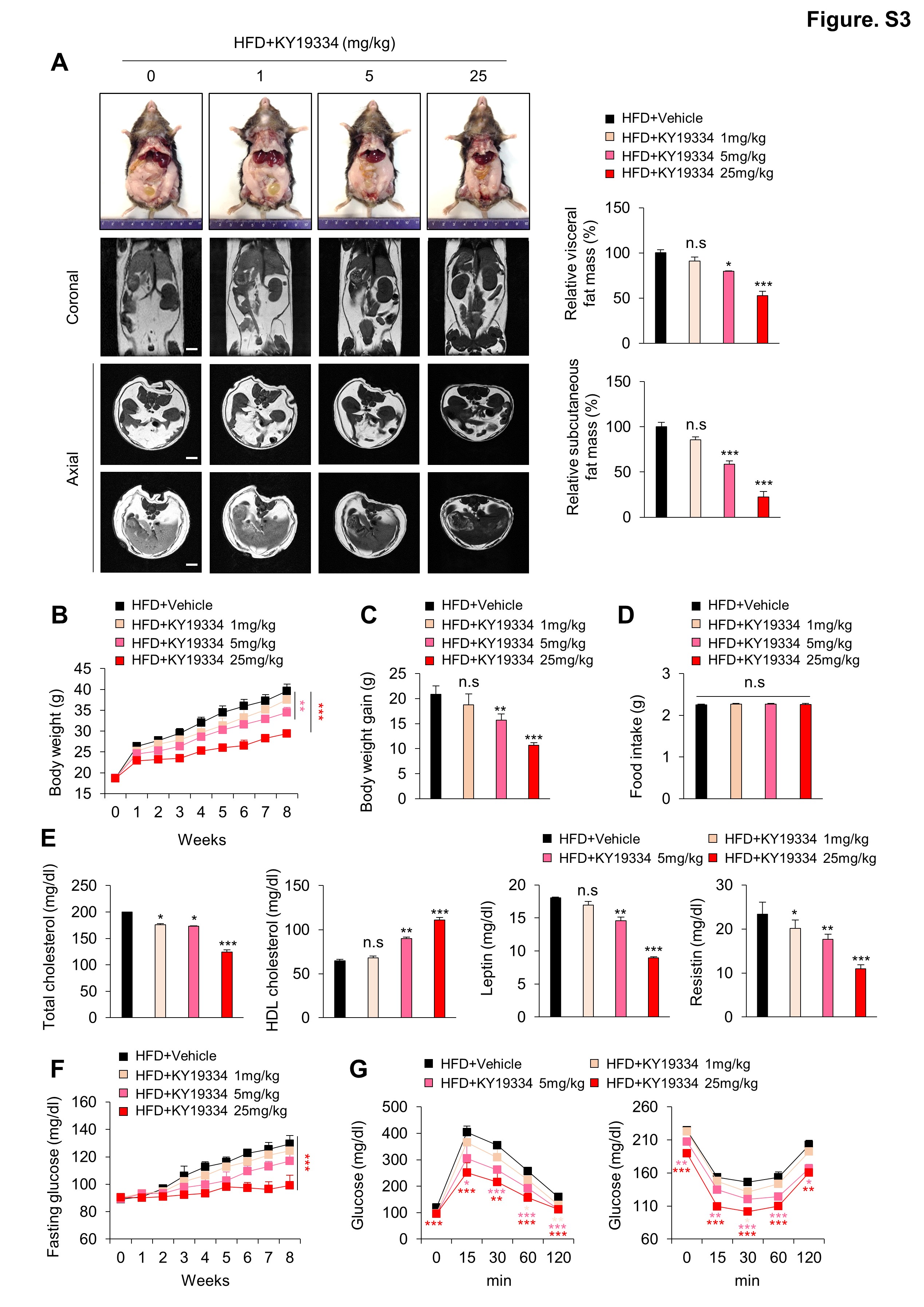


**Supplementary Fig. 3. KY19334 treatment dose-dependently reduces obesity and insulin resistance.** C57BL/6 mice fed the HFD were orally administered KY19334 at concentrations of 1, 5, and 25 mg/kg/d for 8 weeks (*n* = 8 per group). (**A**) Representative photographs and MR images of mice (top panel). Visceral and subcutaneous fat are quantified (bottom panel). (**B**) Body weight changes. (**C**) Body weight gain. (**D**) Daily food intake during all study weeks. (**E**) Plasma concentration of total cholesterol, HDL-cholesterol, leptin, and resistin in the overnight fasted state. (**F**) Fasting glucose. (**G**) Glucose and insulin tolerance test. Scale bar, 100 µm. Results are expressed as mean ± SD, *n* = 3 per group, **P* < 0.05, ***P* < 0.01, ****P* < 0.001, n.s indicates non-significance compared with HFD-fed vehicle group.


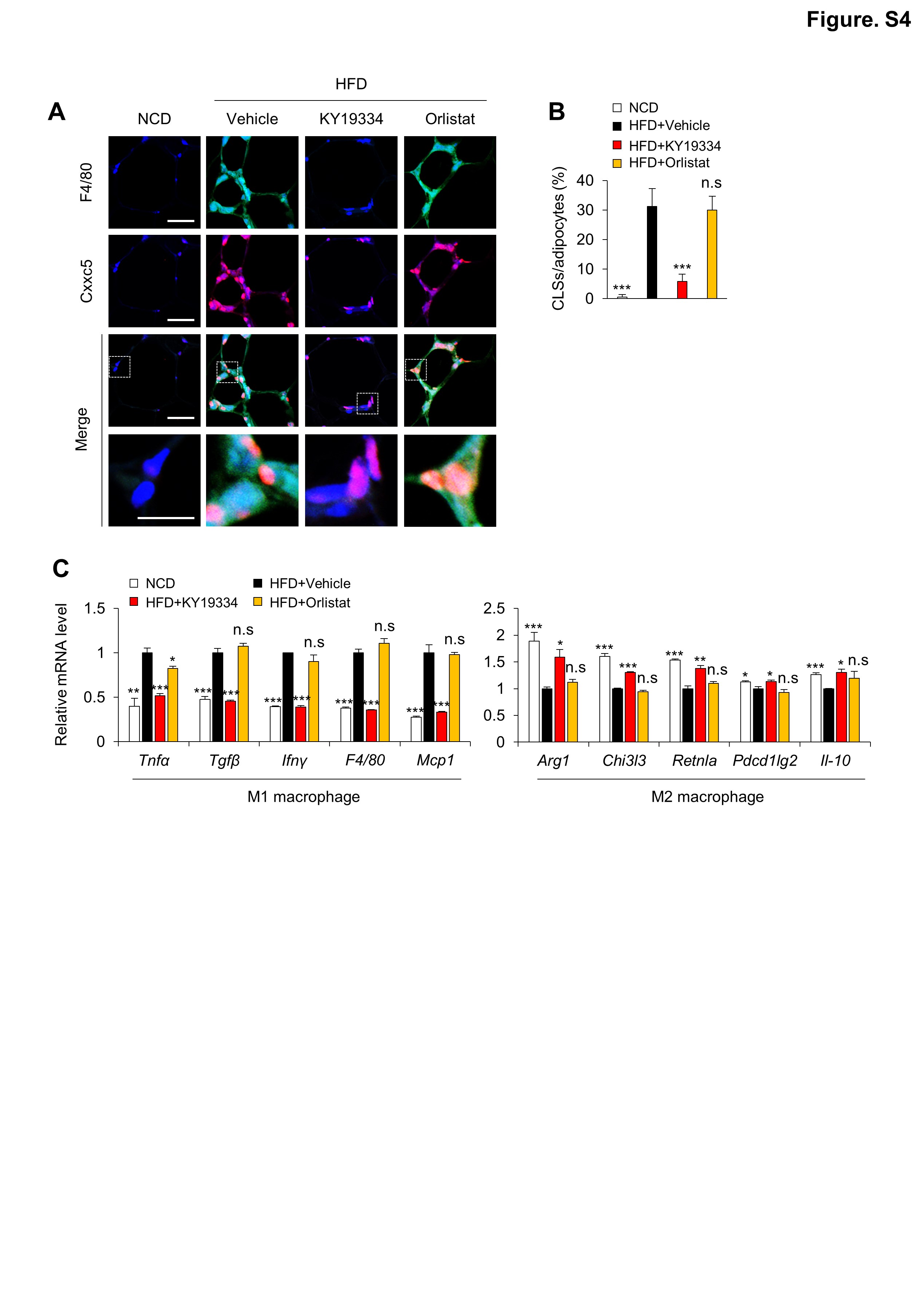


**Supplementary Fig. 4. KY19334 reduces inflammation in scWAT.** The data shown were obtained from the same samples that examined in Fig. 4. (**A**) Representative IHC images for F4/80 and Cxxc5 in the scWAT. (**B**) Quantitative the percentage of crown-like structures (CLSs) per adipocytes on histological section. (**C**) Relative expression levels of marker genes for M1 and M2 macrophage. Scale bar, 100 µm. Results are expressed as mean ± SD., *n* = 3 per group, **P* < 0.05, ***P* < 0.01, ****P* < 0.001, n.s indicates non-significance with HFD-fed vehicle group.

**
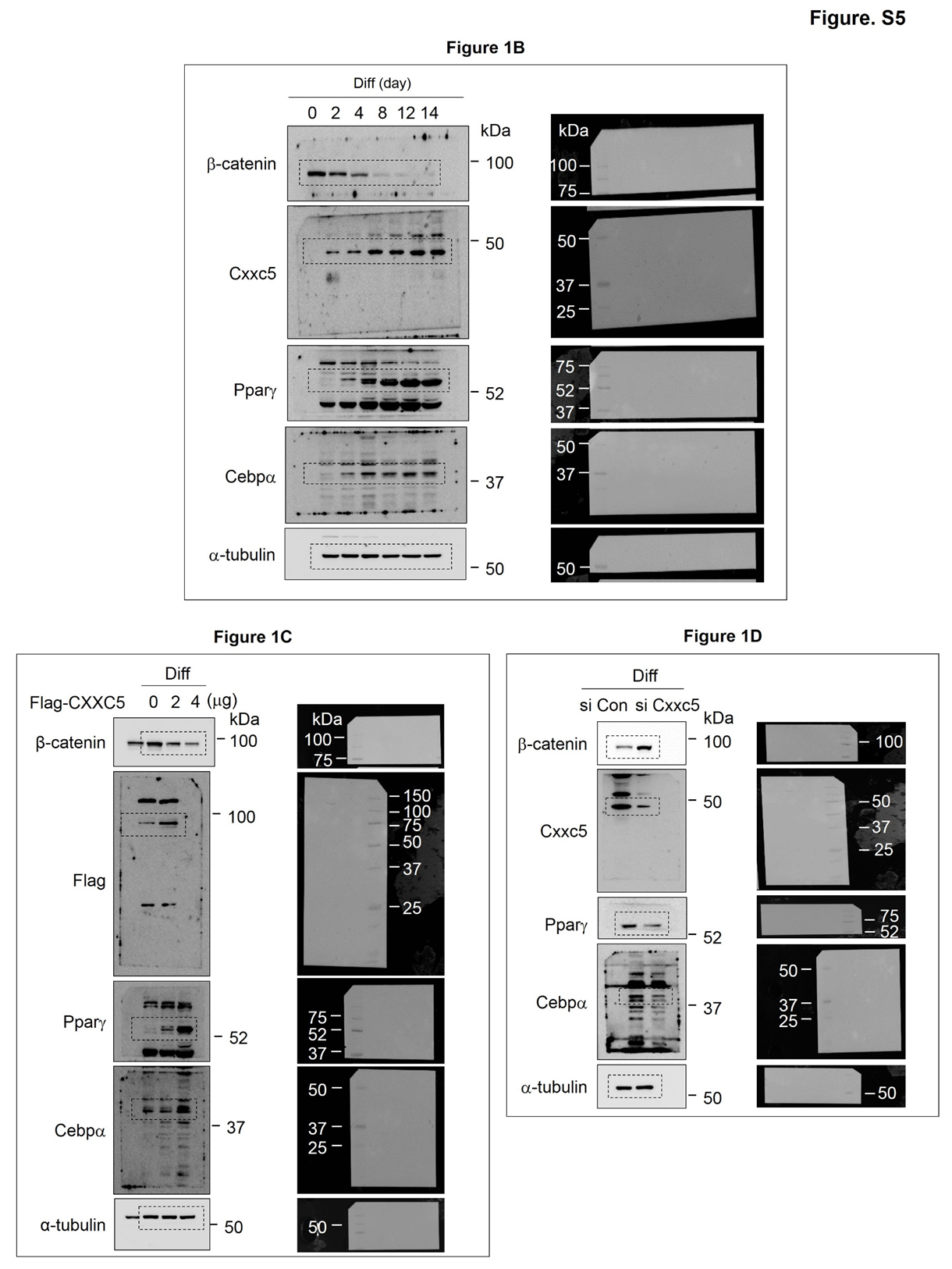
**

**
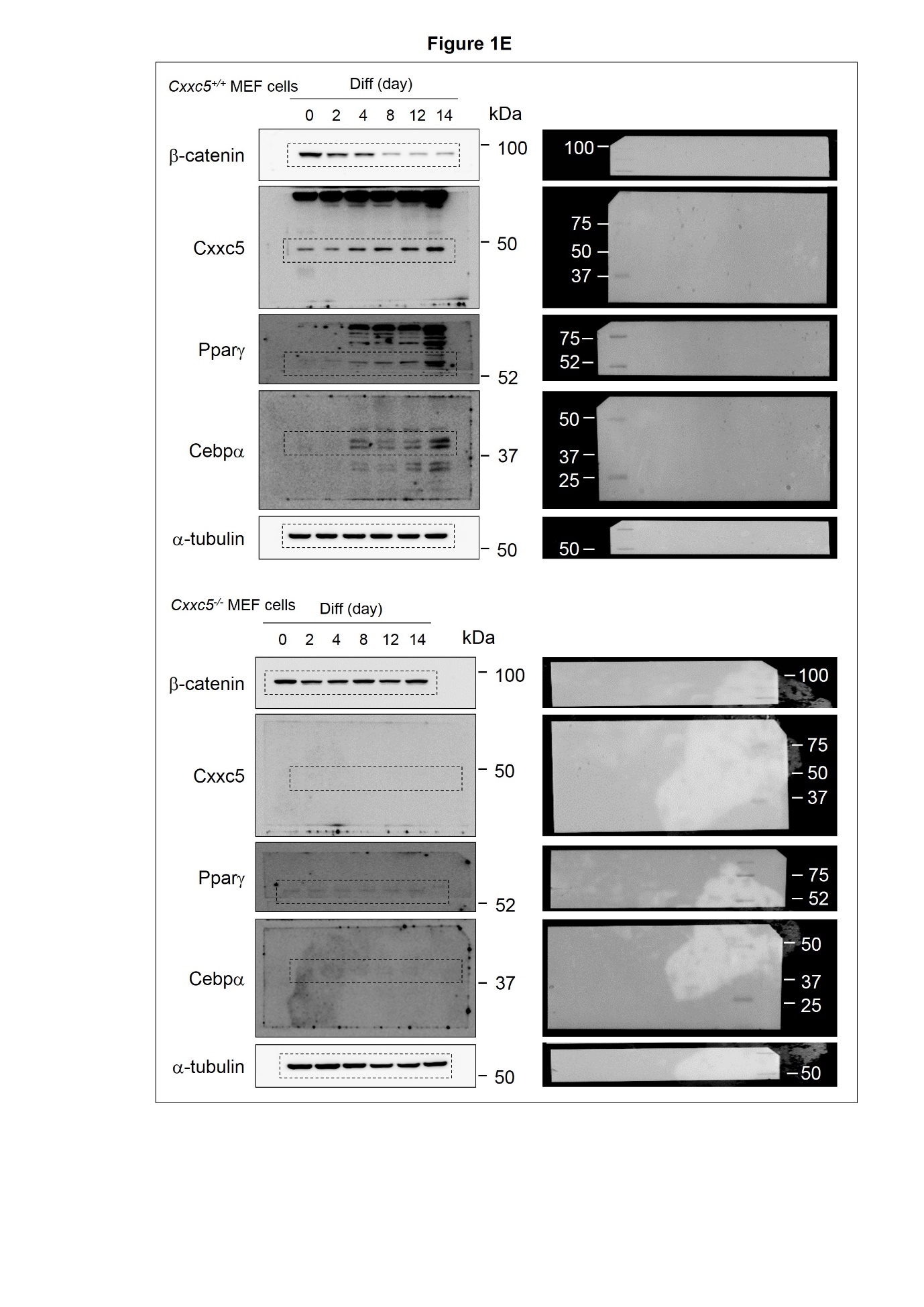
**

**
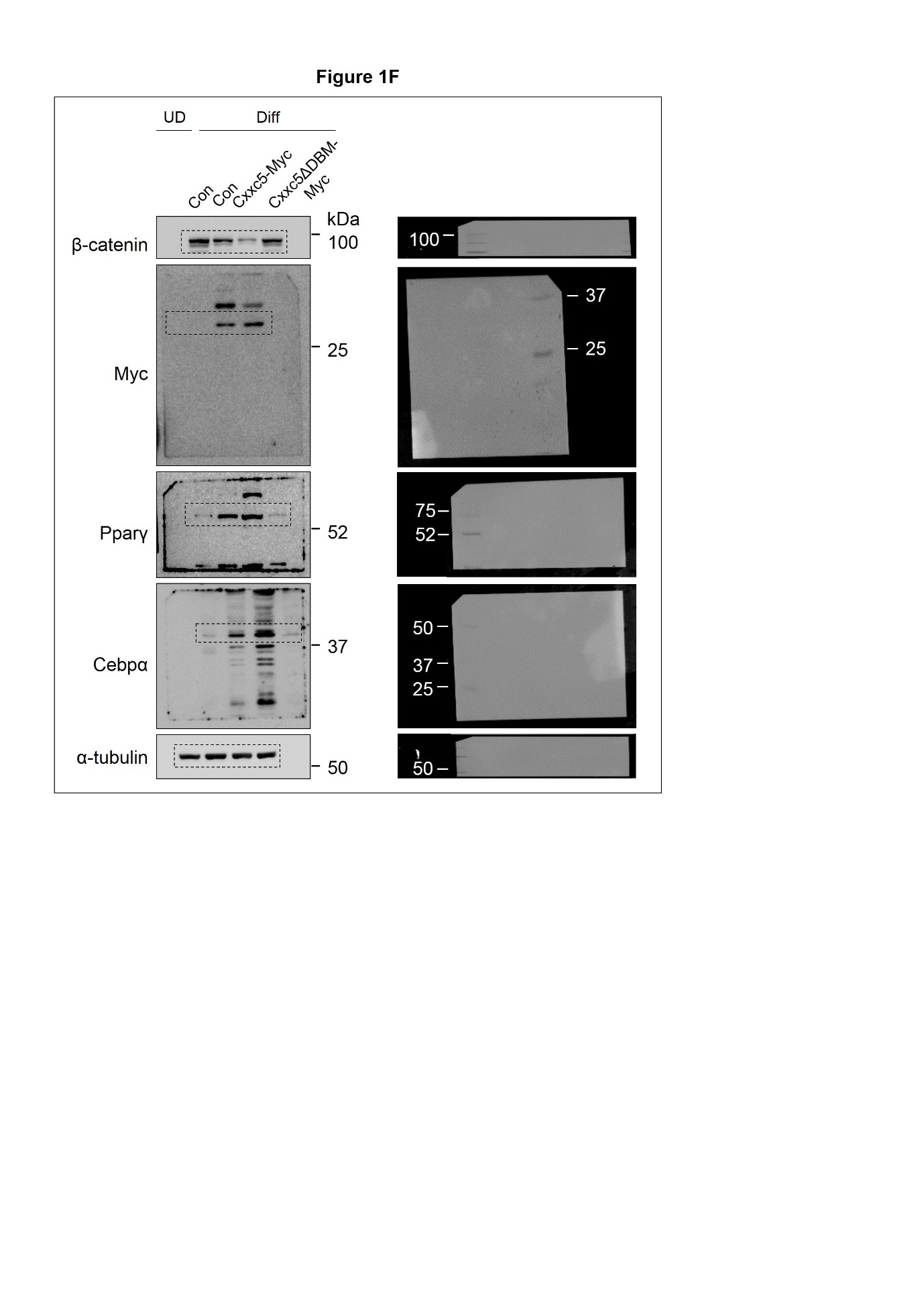
**

**Supplementary Fig. 5. Uncropped blots for Figure 1.** Bio-Rad marker was used as a protein size marker. Alpha-tubulin was used as loading control. Relevant bands are indicated with dotted line.

**
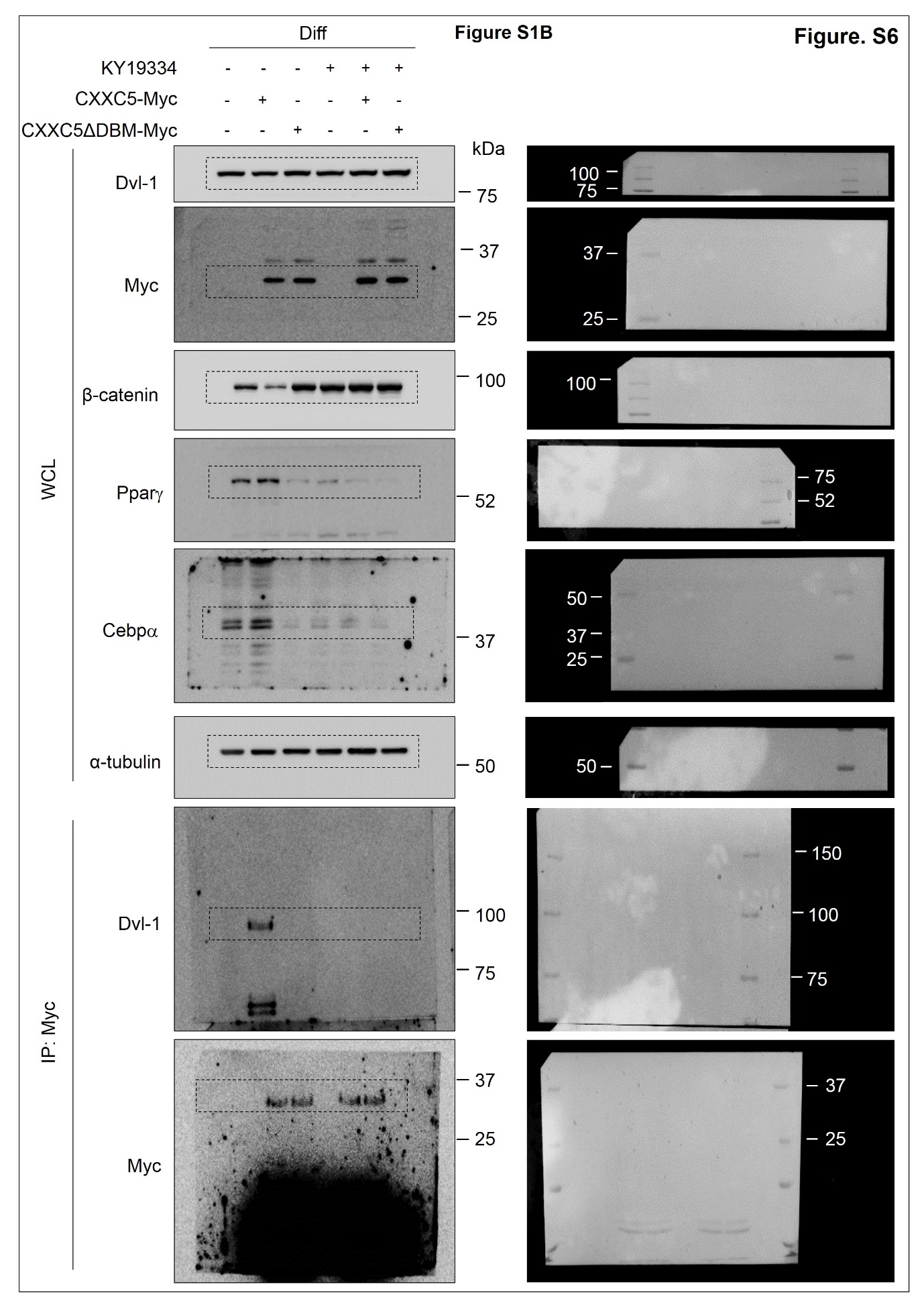
**

**Supplementary Fig. 6. Uncropped blots for Supplemental Fig. 1B.** Bio-Rad marker was used as a protein size marker. Alpha-tubulin was used as loading control. Relevant bands are indicated with dotted line.

**Supplementary Table1. Sequences of the real-time PCR primers used in the study.**

| *Gene* | Forward | Reverse |
| --- | --- | --- |
| *Tcf7l2* | 5’-TGTGTACCCAATCACGACAGGAG-3’ | 5’-GATTCCGGTCGTGTGCAGAG-3’ |
| *Glp-1* | 5’-CCTGGTTGGTATCCCGGGA-3’ | 5’-CCGCTTCAGCTGAAGTCGCA-3’ |
| *Axin2* | 5’-TGGAGAGTGAGCGGCAGAGC-3’ | 5’-TGGAGACGAGCGGGCAGA-3’ |
| *Fosl1* | 5’-AACCGGAGGAAGGAACTGAC-3’ | 5’-CTGCAGCCCAGATTTCTCA-3’ |
| *Wisp1* | 5’-ATCGCCCGAGGTACGCAATAGG-3’ | 5’-CAGCCCACCGTGCCATCAATG-3’ |
| *Pparγ* | 5’-TGTGGGGATAAAGCATCAGGC-3’ | 5’-CCGGCAGTTAAGATCACACCTAT-3’ |
| *Cecpα* | 5’-GGTGGACAAGAACAGCAACGA-3’ | 5’-TGTCCAGTTCACGGCTCAGCT-3’ |
| *aP2* | 5’-ACACCGAGATTTCCTTCAAACTG-3’ | 5’-CCATCTAGGGTTATGATGCTCTTCA-3’ |
| *Lpl* | 5’-CAGAGTTTGACCGCCTTCC-3’ | 5’-AATTTGCTTTCGATGTCTGAGAA-3’ |
| *Srebp1* | 5’-GGAGCCATGGATTGCACATT-3’ | 5’-GGCCCGGGAAGTCACTGT-3’ |
| *Fas* | 5’-GCGATGAAGAGCATGGTTTAG-3’ | 5’-GGCTCAAGGGTTCCATGTT-3’ |
| *Scd-1* | 5’-CTGTACGGGATCATACTGGTTC-3’ | 5’-GCCGTGCCTTGTAAGTTCTG-3’ |
| *Acc* | 5’-CCTCCGTCAGCTCAGATACA-3’ | 5’-TTTACTAGGTGCAAGCCAGACA-3’ |
| *Tnfα* | 5’-CGGAGTCCGGGCAGGT-3’ | 5’-GCTGGGTAGAGAATGGATCA-3’ |
| *Tgfβ* | 5’-TGACGTCACTGGAGTTGTACGG-3’ | 5’-GGTTCATGTCATGGATGGTGC-3’ |
| *Ifnγ* | 5’-TCAAGTGGCATAGATGTGGAAGAA-3’ | 5’-TGGCTCTGCAGGATTTTCATG-3’ |
| *F4/80* | 5’-CTTTGGCTATGGGCTTCCAGTC-3’ | 5’-GCAAGGAGGACAGAGTTTATCGTG-3’ |
| *Mcp1* | 5’-ACTGAAGCCAGCTCTCTCTTCCTC-3’ | 5’-TTCCTTCTTGGGGTCAGCACAGAC-3’ |
| *Arg1* | 5’-CTCCAAGCCAAAGTCCTTAGAG-3’ | 5’-GGAGCTGTCATTAGGGACATCA-3’ |
| *Chi3l3* | 5’-CAGGTCTGGCAATTCTTCTGAA-3’ | 5’-GTCTTGCTCATGTGTGTAAGTGA-3’ |
| *Retnla* | 5’-CCAATCCAGCTAACTATCCCTCC-3’ | 5’-ACCCAGTAGCAGTCATCCCA-3’ |
| *Pdcd1lg2* | 5’-TTGTCGGTGTGATTGGCTTC-3’ | 5’-AAAAGGCAGCACACAGTTGC-3’ |
| *Il-10* | 5’-GCTATGCTGCCTGCTCTTACT-3’ | 5’-CCTGCTGATCCTCATGCCA-3’ |
| *Ucp1* | 5’-AGGCTTCCAGTACCATTAGGT-3’ | 5’-CTGAGTGAGGCAAAGCTGATTT-3’ |
| *Pgc-1α* | 5’-AGCCGTGACCACTGACAACGAG-3’ | 5’-GCTGCATGGTTCTGAGTGCTAAG-3’ |
| *Prdm16* | 5’-CCACCAGCGAGGACTTCAC-3’ | 5’-GGAGGACTCTCGTAGCTCGAA-3’ |
| *Elovl3* | 5’-TTCTCACGCGGGTTAAAAATGG-3’ | 5’-GAGCAACAGATAGACGACCAC-3’ |
| *Cox8b* | 5’-GAACCATGAAGCCAACGACT-3’ | 5’-GCGAAGTTCACAGTGGTTCC-3’ |
| *Pparδ* | 5’-TCCATCGTCAACAAAGACGGG-3’ | 5’-ACTTGGGCTCAATGATGTCAC-3’ |
| *Cd137* | 5’-CCTTGCAGGTCCTTACCTTGT-3’ | 5’-GTTGCTTGAATATGTGGGGGA-3’ |
| *Tmem26* | 5’-ATGGTGCATTTCAAGAAGCC-3’ | 5’-GCTCACCCTCAAGTTCAAGC-3’ |
| *Tbx1* | 5’-CTGTGGGACGAGTTCAATCAG-3’ | 5’-TTGTCATCTACGGGCACAAAG-3’ |
| *Irs1* | 5’-GGACTTGAGCTATGACACGGG-3’ | 5’-GCCAATCAGGTTCTTTGTCTGAC-3’ |
| *G6pc* | 5’-GTCGTGGCTGGAGTCTTG-3’ | 5’-CGGAGGCTGGCATTGTAG-3’ |
| *Pepck* | 5’-ATCTCCTTTGGAAGCGGATATG-3’ | 5’-CGCAACGCAAAGCATTTCTT-3’ |
| *Pck1* | 5’-GGTATTGAACTGACAGACTC-3’ | 5’-CCAGTTGTTGACCAAAGG-3’ |
| *Fbp1* | 5’-GTAACATCTACAGCCTTAATGAG-3’ | 5’-CCAGAGTGCGGTGAATATC-3’ |
